# Photodynamic Therapy: A Rising Star in Pharmaceutical Applications

**DOI:** 10.3390/pharmaceutics16081072

**Published:** 2024-08-16

**Authors:** Eduard Preis, Udo Bakowsky

**Affiliations:** Department of Pharmaceutics and Biopharmaceutics, University of Marburg, Robert-Koch-Str. 4, 35037 Marburg, Germany

## 1. Introduction

**Figure 1:**
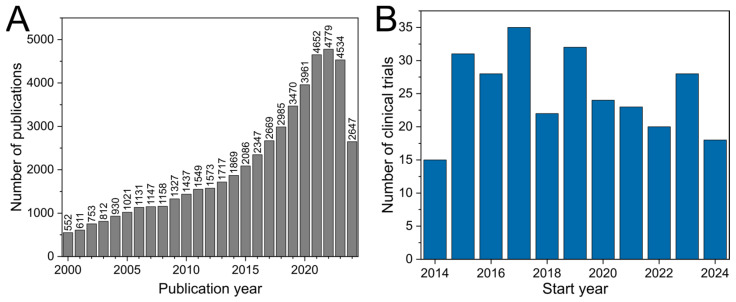
Bar chart depicting (**A**) the number of publications per year from 2000 to 2024, resulting from the search for “photodynamic therapy” from https://www.webofscience.com/wos/woscc/basic-search [1] (accessed on 8 August 2024) and (**B**) the number of clinical trials started each year from 2014 to 2024, resulting from the search for “photodynamic therapy” from https://clinicaltrials.gov [2].

The interest in photodynamic therapy (PDT) has increased remarkably over the past years, with over 75% of all related articles published after 2010 (Figure 1). However, comparing the number of publications (35999 since 2014 [1]) to clinical trials (276 since 2014 [2]), one might think its transition into clinical practice is still modest.

With this Special Issue of *Pharmaceutics*, we would like to encourage researchers to look into photodynamic therapy by highlighting the exceptional work of scientists working in this field. These papers cover the following two main areas: cancer and antimicrobial treatment.

## 2. Photodynamic Therapy

In a phase I/II study, Christensen et al. (contribution 1) investigated the application of 5-aminolevulinic acid (5-ALA), a precursor for the photoactive protoporphyrin IX (PPIX), in extracorporeal photopheresis (ECP) for treating cutaneous T-cell lymphoma (CTCL), focusing on safety and tolerability. In brief, the treatment comprised a blood collection, leukocyte separation, and treatment with 5-ALA for 1 h before UVA light exposure. It showed a 53% reduction in skin involvement and a 50% reduction in pruritus, with no significant changes in vital signs and only mild adverse events.

Reburn et al. (contribution 2) also relied on the precursor 5-ALA in their study. However, their main goal was to increase its conversion to PPIX using an iron-chelating agent (CP94) ester-linked to 5-ALA. This novel iron-chelating prodrug, AP2-18, increased PPIX fluorescence significantly compared to other established PPIX precursors and showed promise in enhancing the efficacy of PDT.

In their study, Roschenko et al. (contribution 3) successfully prepared curcumin-loaded lipid-coated polymeric nanoparticles (CUR-LCNPs) and tested their photodynamic efficacy against HPV^pos^ and HPV^neg^ head and neck squamous cell carcinoma cell lines. Although the HPV status usually leads to significantly different outcomes in other treatment options, PDT with CUR-LCNPs showed similar reductions in cell viability, still exhibiting high biocompatibility.

Whereas most work on PDT relies on direct effects, like reducing cell viability, and establishes the cause by assessing which mechanism type is predominant (Type I or Type II), the review article by Chou et al. (contribution 4) highlights a commonly neglected aspect of PDT, the immunological effects. They elaborate on the effectiveness of PDT-induced anti-tumor immunity and discuss influence factors, such as the localization of photosensitizers (PS), used concentration, light fluence rate, oxygen concentration, and immune response capabilities. Additionally, they present insights into combination therapy that can lead to synergistic effects.

## 3. Antimicrobial Photodynamic Therapy

Kaur et al. (contribution 5) explored the use of ruthenium-based photosensitizers encapsulated in enzyme-degradable nanocarriers consisting of PEG_114_-b-PLA_143_ and PEG_114_-b-PLA_499_ polymers. They saw an increase in fluorescence after incubation with proteinase K, hinting at enzymatic degradation, and significantly improved the antimicrobial effect, achieving up to a 4.9 log reduction in bacterial activity.

In their review article, Passaglia et al. (contribution 6) present the multifunctionality of phosphorene, a 2D nanomaterial derived from black phosphorus. Among other areas, it can be used in biomedical applications, and because of its intrinsic photoactivity, it is suitable for photothermal and photodynamic therapy.

The systematic review by Schweigert et al. (contribution 7) shows that the effectiveness of PDT varies depending on the PS, and choosing the appropriate PS is crucial for personalizing peri-implantitis treatment. Further research is needed to standardize protocols and evaluate their long-term effectiveness.

## 4. Conclusions/Future Directions

PDT/aPDT is still limited by a multitude of influencing factors. Whereas the efficacy of antibiotics or chemotherapeutics primarily relies on the type and concentration of the substance, proper photodynamic dosimetry has to additionally consider radiant exposure and local oxygen concentration. Thus, more information about the targeted tissue and interpersonal differences is necessary to accomplish good outcomes. More robust and detailed work from cross-functional research groups will bring forth appropriate tools to improve PDT applicability and clinically approved treatment options.

## Data Availability

No new data were created or analyzed in this study. Data sharing is not applicable to this article.
